# Time to broaden the scope of research on anticipatory behavior: a case for the role of probabilistic information

**DOI:** 10.3389/fpsyg.2015.01518

**Published:** 2015-10-02

**Authors:** Rouwen Cañal-Bruland, David L. Mann

**Affiliations:** Department of Human Movement Sciences, MOVE Research Institute Amsterdam, VU University AmsterdamAmsterdam, Netherlands

**Keywords:** anticipation, prediction, judgment, decision-making, sport expertise, social interaction

Over the past four decades we have seen a dramatic improvement in our understanding of the processes that underpin the anticipatory behavior of skilled performers in domains such as sport. Early research by Jones and Miles (1978) and Salmela and Fiorito (1979) inspired and fuelled the research of today's leaders in the field such as Abernethy (1990), Savelsbergh et al. (2002), and Williams and Davids (1998), and many of us who follow in their footsteps. Originally, the key question driving this research was whether skilled performers of temporally constrained sport tasks (e.g., returning a tennis serve or hitting a baseball) are better than less-skilled performers in their ability to make use of kinematic information from an opponent's action. After confirming the expert advantage in anticipation, research then focused on identifying the kinematic sources of information that underpin the superior anticipatory behavior. This research made use of a variety of experimental paradigms including temporal and spatial occlusion techniques, point-light displays, and gaze tracking (for a review see Mann and Savelsbergh, 2015). Since the 1970s an impressive body of empirical data has been generated that has led to useful practical outcomes for the individual sports examined, and that at the same time have resulted in more generalizable findings across sports, thereby generating a better understanding of the mechanisms underlying expert anticipation. As a case in point, it is now generally accepted that experts tend to make use of fewer fixations of longer duration when trying to predict the outcome of an opponent's action (for a meta-analysis supporting this conclusion, see e.g., Mann et al., 2007). There can be little doubt that this research focusing on the kinematic sources of information that facilitate the prediction of action outcomes has proven to be very insightful and important for furthering our understanding of expert anticipation in sports (Abernethy, 1990; Williams and Davids, 1998). Likewise, this research has been crucial for generating evidence-based recommendations for the best means to train anticipatory skill. However, here we call for a broadening in the scope of anticipation research in an attempt to further improve and enrich our understanding of expert anticipation in sport. This call is based on the high proportion of studies performed examining anticipatory behavior on the basis of kinematic sources of information, yet a relative paucity in studies that take into consideration the influence of broader situational or contextual (non-kinematic) sources of information.

As early as in the late 1970s researchers identified that anticipatory behavior may at least in part be informed by probabilistic information that is independent of the observed movement, and hence also independent of the visual information that can be picked up from such movements (e.g., Alain and Girardin, 1978; Alain and Proteau, 1980). It was Abernethy et al. (2001) who—more than 20 years after the original observations had been published—revisited how other non-kinematic, contextual information may contribute and influence how experts anticipate action outcomes. In their seminal paper, Abernethy et al. (2001) showed that probabilistic information that they coined *situational probabilities* could be used to anticipate action outcomes in the absence of any movement information from the opponent (in that case by evaluating the court position of the opponent in squash). Since then, only recently have a handful of studies started to systematically examine the contribution of situational probability (or *contextual*) information to anticipatory behavior. This includes the impact of probabilistic information such as playing patterns related to the game score (Farrow and Reid, 2012); exposure to an individual's action preferences (Navia et al., 2013; Mann et al., 2014); exposure to previous outcome sequences (Loffing et al., 2015); how an opponent's court position interacts with kinematically-driven judgments (Loffing and Hagemann, 2014); and how contextual information influences both gaze behavior (McRobert et al., 2011) and the cognitive processes underpinning anticipatory skill (Murphy et al., 2015). Moreover, it has been shown that the anticipatory behavior of skilled performers is influenced by their assessment of the relative costs and benefits of responding or not responding (Cañal-Bruland and Schmidt, 2009; Cañal-Bruland et al., 2015). Further, work on the use of simple heuristics also indicates that experts tend to use various sources of information to make fast judgments under conditions of uncertainty that in sports include situations which require the initiation of action responses even before reliable visual information is available (Raab, 2012; de Oliveira et al., 2014). Together, these findings strongly support the idea that factors other than visual information conveyed in the observed kinematics do also play a significant role in successful anticipatory behavior.

Given that these recent studies have highlighted a surprisingly large role for contextual information in supporting anticipatory behavior, we advocate that more research is needed in this area to bring us closer to the original aim, namely, to understand the superior anticipatory skill of experts. To reach this ultimate objective, in our view, research is needed to identify: (1) the contextual (non-kinematic) sources of information that influence anticipatory behavior; (2) how skilled performers combine these non-kinematic contextual sources of information with (i) each other and (ii) with real-time (kinematic) information from an opponent's actions; and (3) how the way that the information is combined is shaped by the circumstances in which the behavior is performed.

## Step 1: The contextual sources of information

Ideally we need to work toward an understanding of the different sources of contextual information that can underpin anticipatory behavior. This is not a simple process though as the contextual information will necessarily be sport-specific. For instance, an opponent's court position may provide useful information when anticipating an opponent's shot in squash, but not for a soccer goalkeeper seeking to save a penalty. Interviews with athletes and coaches may be useful for the development of empirically verifiable sport-specific models that outline the different sources of information that skilled athletes rely on (e.g., Greenwood et al., 2014; Schläppi-Lienhard and Hossner, 2015). As our understanding of the different sources of information grows, hopefully we can cultivate a more general conceptualisation of the contextual information used across tasks to help predict the behavior of players in real-life scenarios.

## Step 2: Combining sources of contextual information

In any particular scenario there are likely to be a number of different sources of contextual information, in addition to the kinematic movement pattern of the opponent. As a result, performers will need to collectively account for this information to guide their anticipatory response. It will be useful to develop an understanding of how the different sources of contextual and kinematic information influence anticipatory behavior; this is certainly not a trivial endeavor as there are various ways how such an influence might be effectuated. It could be that motor behaviors are generated on the basis of *the most prominent* source of contextual or kinematic information; an additive combination of the informational sources; or through the interaction of the informational sources. Contextual information is typically picked-up well before kinematic information becomes available and so it would be interesting to know when and how the different sources of information enter anticipatory behavioral processes as both are likely to function on different time scales.

Moreover, for a given scenario it could be that some performers adopt different strategies to others. For instance, soccer penalty takers frequently adopt “keeper-dependent” or “keeper-independent” strategies where they respectively take into account or ignore the movements of the goalkeeper when deciding where to direct their kick (Kuhn, 1988). In this case those adopting a keeper-dependent strategy are likely to prioritize kinematic information whereas those using a keeper-independent strategy may rely more heavily on contextual information (e.g., about the goalkeeper's preferences for direction of dive).

## Step 3: How circumstances shape the use of contextual information

It seems reasonable to expect that the manner in which informational sources are combined to produce a motor response may be influenced by the specific circumstances in which the action takes place. In particular, contextual information is likely to become increasingly important when the temporal demands associated with the task increase. As a case in point, a tennis player may not need to rely very heavily on contextual or even kinematic information when returning an opponent's ground stroke (Triolet et al., 2013), yet the contribution of that information is likely to be much more important if attempting to return an opponent's volley from the net. Another example is that the manner in which the contextual information is acquired could influence the usefulness of that information. It has become increasingly common for professional sporting teams to employ performance analysts to inform players about the behavioral patterns of their opponents. By raising explicit awareness of this information it could be that the athlete is more likely to generate their responses on the basis of that information and ignore (or rely less on) the remaining informational sources that they might typically rely on (Mann et al., 2014; Gray, 2015). A model of anticipatory behavior will need to account for the ways in which behavior is observed to change commensurate with alterations in the circumstances in which the task is performed.

We hope that a solid understanding of contextual informational sources will lead to the development of an overarching theoretical framework that can predict and explain anticipatory behavior, and therefore will provide empirically testable and falsifiable hypotheses to guide future work. To stimulate this process, here we have highlighted the skewed distribution of research in favor of the pick-up of advance visual information at the expense of other factors such as situational probability and contextual information. We consequently call for a broadening of the scope of research on anticipatory behavior and hope that many young and not so young researchers join us in this endeavor in order to help improve our understanding of anticipation and how it facilitates motor performance.

### Conflict of interest statement

The authors declare that the research was conducted in the absence of any commercial or financial relationships that could be construed as a potential conflict of interest.
